# Additively Manufactured Dual‐Faced Structured Fabric for Shape‐Adaptive Protection

**DOI:** 10.1002/advs.202301567

**Published:** 2023-05-10

**Authors:** Yuanyuan Tian, Kaijuan Chen, Han Zheng, Devesh R. Kripalani, Zhuohong Zeng, Asker Jarlöv, Jiayao Chen, Lichun Bai, Adrian Ong, Hejun Du, Guozheng Kang, Qihong Fang, Lihua Zhao, H. Jerry Qi, Yifan Wang, Kun Zhou

**Affiliations:** ^1^ HP‐NTU Digital Manufacturing Corporate Lab Nanyang Technological University 50 Nanyang Avenue Singapore 639798 Singapore; ^2^ Singapore Centre for 3D Printing School of Mechanical and Aerospace Engineering Nanyang Technological University 50 Nanyang Avenue Singapore 639798 Singapore; ^3^ State Key Laboratory of Advanced Design and Manufacturing for Vehicle Body Hunan University Changsha Hunan 410082 China; ^4^ School of Traffic and Transportation Engineering Central South University Changsha Hunan 410083 China; ^5^ School of Mechanics and Aerospace Engineering Southwest Jiaotong University Chengdu Sichuan 610031 China; ^6^ 3D Lab HP Labs HP Inc. Palo Alto CA 94304 USA; ^7^ The George W. Woodruff School of Mechanical Engineering Georgia Institute of Technology Atlanta GA 30332 USA

**Keywords:** additive manufacturing, deformation recovery, dual‐faced structured fabric, energy absorption, shape‐adaptive protection

## Abstract

Fabric‐based materials have demonstrated promise for high‐performance wearable applications but are currently restricted by their deficient mechanical properties. Here, this work leverages the design freedom offered by additive manufacturing and a novel interlocking pattern to for the first time fabricate a dual‐faced chain mail structure consisting of 3D re‐entrant unit cells. The flexible structured fabric demonstrates high specific energy absorption and specific strength of up to 1530 J kg^−1^ and 5900 Nm kg^−1^, respectively, together with an excellent recovery ratio of ≈80%, thereby overcoming the strength–recoverability trade‐off. The designed dual‐faced structured fabric compares favorably against a wide range of materials proposed for wearable applications, attributed to the synergetic strengthening of the energy‐absorbing re‐entrant unit cells and their unique topological interlocking. This work advocates the combined design of energy‐absorbing unit cells and their interlocking to extend the application prospects of fabric‐based materials to shape‐adaptive protection.

## Graphical abstract

A dual‐faced chain mail structure is fabricated for the first time through leveraging the design freedom offered by additive manufacturing and a novel interlocking pattern. The flexible structured fabric demonstrates high specific energy absorption and specific strength, together with excellent recovery ratio, thereby overcoming the strength–recoverability trade‐off. This work extends the application prospects of fabric‐based materials to shape‐adaptive protection.

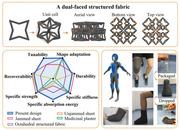

## 1 Introduction

Fabric‐based materials, including woven or interlocking structures, possess an adaptive feature to effectively accommodate complex shapes.^[^ ^1^, ^2^ ^]^ Their functional roles are usually extended to energy harvesting^[^ ^3^ ^]^ and medical treatment^[^ ^4^ ^]^ by the means of multi‐material weaving and double‐faced spraying, respectively. As most of these applications do not have strong requirements on the mechanical properties, the development of load‐bearing fabric‐based materials has received little attention. However, developing a flexible lightweight material with high strength, deformation recoverability, and energy absorption capacity could be an important step toward next‐generation shape‐adaptive protection.

As a promising class of energy‐absorbing materials for applications in protection, lattice structures, based on a periodic arrangement of unit cells in a regular network of struts, possess extraordinary specific strength,^[^ ^5^, ^6^ ^]^ specific energy absorption,^[^ ^7^, ^8^ ^]^ and specific stiffness.^[^ ^9^, ^10^ ^]^ These structures can be designed by various types of structural unit cells, such as face‐centered cubic,^[^ ^11^ ^]^ diamond,^[^ ^12^ ^]^ and re‐entrant^[^ ^13^ ^]^ units, to achieve excellent mechanical properties. Particularly, re‐entrant lattices have demonstrated impressive energy absorption since they typically contract in the directions perpendicular to the loading direction during compression.^[^ ^14^ ^]^ However, once fabricated, the geometric shapes and mechanical properties of these structures are fixed. Thus, the development of adaptive structures with excellent and tunable mechanical properties is highly desirable for shape‐adaptive protection.

By leveraging the superior mechanical properties of lattice materials and the adaptivity of fabric‐based materials, it is possible to realize an adaptive structure with outstanding mechanical properties by interlocking lattice unit cells into a high‐performance chain mail structure. Furthermore, the layer‐by‐layer nature of additive manufacturing, also referred to as 3D printing, offers unparalleled design freedom and allows for the fabrication of structures with a unique combination of delicate geometries and outstanding material properties. Recent work has successfully combined the features of fabric‐based materials and lattice structures utilizing additive manufacturing to obtain a structured fabric with tunable bending modulus.^[^ ^15^ ^]^ However, it has been recognized that the mechanical properties of this structured fabric are still deficient for high‐performance wearable applications, which require high specific strength and energy absorption.

In this work, we developed a dual‐faced structured fabric consisting of 3D re‐entrant unit cells interlocked in a novel chain mail structure with different unit cell arrangements on its top and bottom surfaces. The synergetic strengthening of the energy‐absorbing unit cells and their unique topological interlocking, that is, the intruding vertical struts and the interaction between the horizontal struts in adjacent unit cells, results in a structured fabric with high specific strength and specific energy absorption. The distortion‐induced deformation of unit cells prevents catastrophic failure of the dual‐faced structured fabric and promotes good deformation recoverability. We found that the specific strength and specific energy absorption can be as high as 5900 Nm kg^−1^ and 1530 J kg^−1^, respectively, when applying a small vacuum confining pressure of 90 kPa around the boundaries of the designed structured fabric, which also allows the structure to maintain a high deformation recovery ratio, thereby overcoming the strength–recoverability trade‐off. Additionally, by only applying the vacuum confining pressure of 90 kPa, the specific bending modulus can be improved by up to 67 times which is much higher than what has been reported so far, ascribed to not only the vacuum confining effect but also the structure's dual‐faced feature. The present design would broaden the applications of fabric‐based materials to shape‐adaptive protection such as light‐weight body armor, protective packaging, and multifunctional medical support.

## 2 Results and Discussion

### 2.1 Fabrication and Characterization

**Figure 1:**
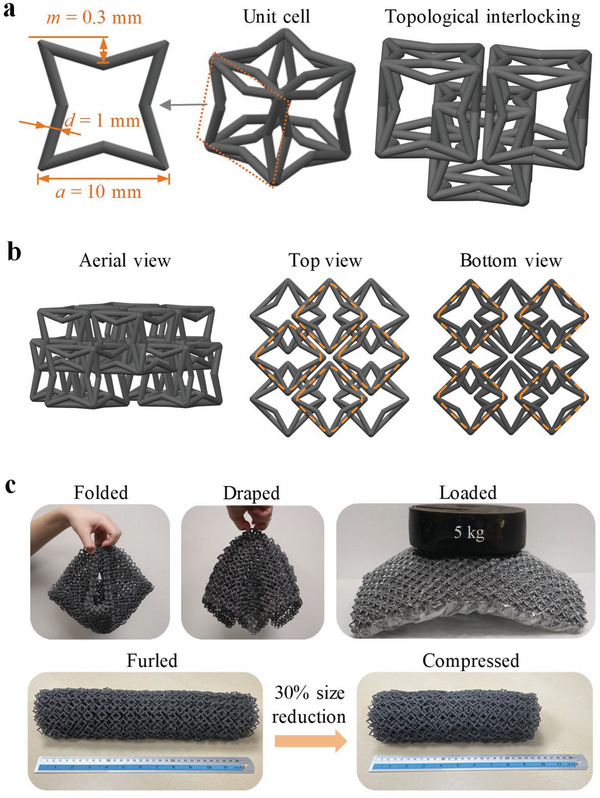
Design and prototype of the dual‐faced structured fabric. a) Schematic illustration of the 3D re‐entrant unit cell in the dual‐faced structured fabric and its topological interlocking. b) Three views of the structured fabric showing the different unit cell arrangements in the top and bottom surfaces. c) Different configurations of the structured fabric, indicating that it can be folded, draped, furled, and compressed in its flexible state, and that it can withstand load under vacuum confining pressure.

The proposed structured fabric consists of 3D re‐entrant unit cells topologically interlocked in a staggered chain arrangement, as shown in **Figure** **1**a. The re‐entrant unit cell is defined by three geometric parameters, including the strut diameter *d*, edge length *a*, and re‐entrant distance *m*. Note that large geometric parameters will result in the overlap of cell walls, whereas small parameters may sacrifice the re‐entrant characteristic.

The structured fabric was printed using polyamide 12 (PA12) by Multi Jet Fusion (MJF), a fast‐growing power bed fusion additive manufacturing technique. The printed structure has different unit cell arrangements for the top and bottom surfaces, resulting in a dual‐faced feature (Figure 1b). Hence, the dual‐faced structured fabric has different bending moduli depending on which surface is loaded, resulting in a wider range of bending stiffness to better withstand different bending moments, as will be shown in the subsequent sections. The structured fabric is highly flexible, and can be folded, draped, and furled (Figure 1c). The furled fabric can be further compressed for storage with a final size reduction of 30% (Movie S1, Supporting Information). After a vacuum pressure has been applied, the confined fabric can withstand a load of more than 25 times its own mass (top right panel of Figure 1c).

### 2.2 Mechanical Properties

**Figure 2:**
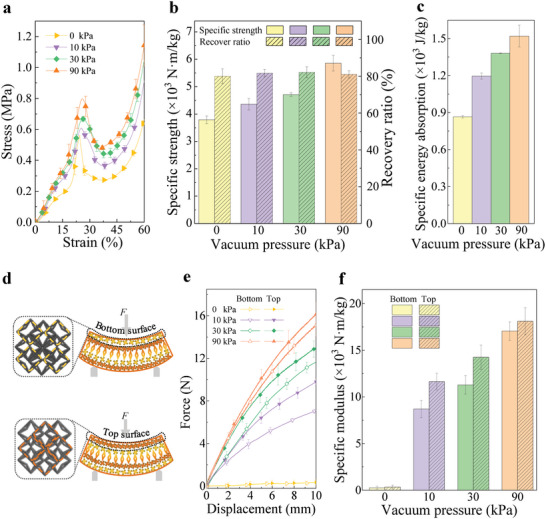
Mechanical properties of the dual‐faced structured fabric. a) Compressive stress–strain curves of the structured fabric under different vacuum confining pressures. b,c) Specific strength, recovery ratio, and specific energy absorption of the structured fabric under different vacuum confining pressures. d) Schematic of bending tests while loading the bottom and top surfaces. e) Force–displacement curves during the bending test of the structured fabric under different vacuum confining pressures while loading the bottom and top surfaces. f) Specific bending modulus of the structured fabric under the corresponding conditions.

To quantitatively explore the mechanical properties of the dual‐faced structured fabric, its specific strength, specific energy absorption, recovery ratio, specific bending modulus, and energy loss coefficient were calculated by performing compression, cyclic compression, and three‐point bending tests (see Experimental Section). **Figure** **2**a gives the compression stress–strain curves of the dual‐faced structured fabric under different vacuum confining pressures. The structure exhibits linear elastic deformation initially, and then goes through a strengthening stage, in which the slopes of the stress–strain curves increase drastically. Finally, the fabric yields at a high stress level and then continues to deform until densification.

Based on the yield strength and energy absorption, the specific strength and specific energy absorption of the structured fabric under different vacuum confining pressures were calculated (see Experimental Section and Section S1, Supporting Information). Figure 2b shows that even in its flexible state without jamming, the structured fabric possesses a high specific strength of 3790 Nm kg^−1^, which exceeds those of some metallic and composite lattice structures (375–2135 Nm kg^−1^).^[^ ^16^, ^17^, ^18^ ^]^ Through vacuum jamming, an even higher specific strength of 5900 Nm kg^−1^ can be obtained. Similarly, its specific energy absorption of 866 J kg^−1^ is also large without jamming (Figure 2c) which is comparable to or even higher than those of many classical energy‐absorbing materials including lattice structures and foam materials (97–1000 J kg^−1^).^[^ ^8^, ^14^, ^16^, ^17^, ^18^, ^19^, ^20^, ^21^, ^22^, ^23^, ^24^, ^25^, ^26^, ^27^ ^]^ Likewise, a much higher specific energy absorption can be achieved (up to 1530 J kg^−1^) under a small vacuum confining pressure (up to 90 kPa was applied). These results indicate that high yield strength and energy absorption capacity can be achieved for the dual‐faced structured fabric, which can be ascribed to the design of the 3D re‐entrant unit cells and their novel topological interlocking, as well as the vacuum jamming effect. The related mechanisms will be analyzed in the subsequent section.

A conflict exists between strength and recoverability in nearly all engineering materials, in particular those with a lattice architecture, restricting the applications of these materials.^[^ ^13^ ^]^ The recovery ratio of the structured fabric was calculated after a compression strain of 70% has been applied under vacuum confining pressures from 0 to 90 kPa (see Experimental Section). The results show that the recovery ratio is about 80% for all pressures studied in the present work while the high yield strength can still be maintained (Figure 2b), revealing that the structured fabric overcomes the strength–recoverability trade‐off. Furthermore, the results from the cyclic compression test indicate good durability and energy restoration capacity of the structured fabric (Section S2, Supporting Information).

Figure 2d presents a schematic of loading the bottom and top surfaces of the dual‐faced structured fabric during the bending tests, while Figure 2e gives their corresponding force–displacement curves under different vacuum confining pressures. Initially, for small displacement, a linear regime is observed, showing an elastic behavior of the structured fabric. Subsequently, a nonlinear response occurs with increasing displacement, implying the onset of frictional sliding and the local rearrangement of the 3D re‐entrant unit cells. Based on the bending modulus determined from the initial linear stage of the force–displacement curve (see Experimental Section), the specific bending modulus of the structured fabric was calculated to investigate the bending performance under different conditions (Figure 2f).

For the case of loading the bottom surface, the specific bending modulus increases significantly when the vacuum confining pressure increases from 0 to 90 kPa, which can be ascribed to the jamming transition triggered by the confining pressure.^[^ ^15^, ^28^, ^29^ ^]^ Apart from the inherent tensile resistance of the structured fabric, the vacuum confining pressure introduces frictional drag between interlocked unit cells. In addition, the specific bending modulus is further increased when the loading surface is changed from the bottom surface to the top surface, implying that the dual‐faced feature of the structured fabric exerts an important influence on the bending modulus. By combining the jamming effect of the vacuum confining pressure with the dual‐faced feature of the structured fabric, the specific bending modulus increases by a factor of over 67, from about 0.27 MPa to about 18.1 MPa, which is a far higher amplification factor than those previously reported in literature.^[^ ^15^, ^28^, ^29^ ^]^

### 2.3 Fundamental Mechanisms

**Figure 3:**
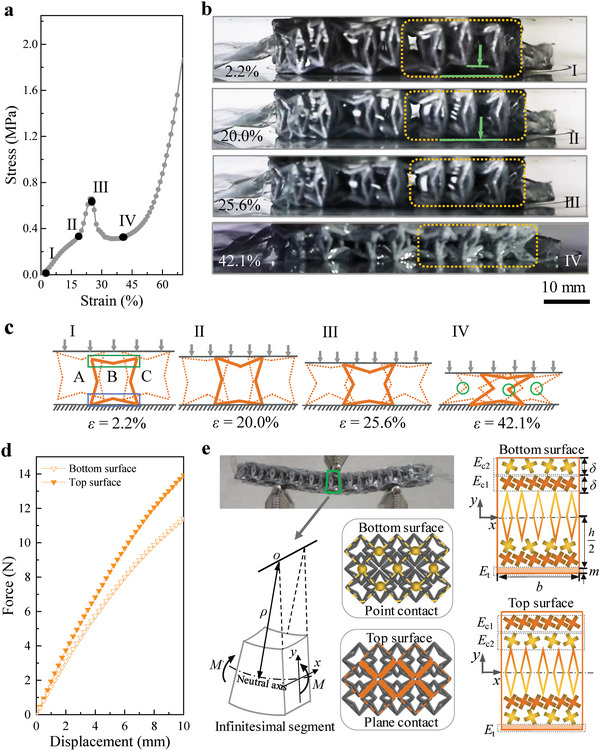
An explanation of the underlying mechanisms contributing to the excellent mechanical properties of the dual‐faced structured fabric. a) Compressive stress–strain curve of the structured fabric under a vacuum confining pressure of 60 kPa. b) Side views showing the still frames from the compression test of the structured fabric sample. Each frame corresponds to a marked point in the stress–strain curve shown in (a). c) Schematic diagram illustrating the deformation response of the structured fabric under a compressive load (indicated by the gray arrows). d) Force–displacement curves during the bending test of the structured fabric while loading the bottom and top surfaces under a vacuum confining pressure of 60 kPa. e) Schematic diagram illustrating the bending deformation of the structured fabric when loading its bottom and top surfaces.

To guide the design of structured fabrics with excellent mechanical properties, it is imperative to reveal the deformation and strengthening mechanisms of the dual‐faced structured fabric during compression. **Figure** **3**a shows that there is an abrupt increment in the measured stress after state II (about 20% strain), which contributes to the high yield strength of the structured fabric. This phenomenon is due to a change in the deformation behavior at state II.

Initially (Figure 3b, state I), the unit cells are only in contact with either the upper or the lower compression platform, as shown in Figure 3c, state I (unit cells A and C have their top surface in contact with the upper platform and unit cell B has its bottom surface in contact with the lower platform). During compression, the deformation of the horizontal struts in contact with the platforms is hindered due to their mutual interaction with adjacent horizontal struts not in contact with the platforms. Consequently, this interaction between the horizontal struts as a result of the novel topological interlocking causes the initial increase in the stress (Figure 3a, from state I to state II).

With increasing strain, the unit cells are compressed until all of them are brought in contact with both the upper and lower compression platforms (Figure 3b II). When the strain exceeds 20% (Figure 3b, from state II to state III), in addition to the interaction between the horizontal struts in adjacent unit cells, the intruding vertical struts provide additional strength to withstand higher loading (Figure 3c, state III). These two effects synergistically contribute to the abrupt increase in the slope of the stress–strain curve (Figure 3a, from state II to state III).

Once the compressive force is sufficiently large to overcome the friction force, the interacting horizontal struts start to slide over each other, thereby causing distortions in the unit cells and the further deformation of the structured fabric (Figure 3b,c, state IV). The distortion‐induced deformation can be attributed to the unique structure of the 3D re‐entrant unit cells (i.e., the intruding struts) in the structured fabric as well as the dual‐faced feature. As shown in Figure 3c, state IV, the horizontal struts on the top surface of the unit cell have a tendency to slide along the downward‐sloping struts of adjacent unit cells (labeled by the green box); while for those on the bottom surface, the sliding occurs along the upward‐sloping struts (labeled by the blue box) and is thus limited during compression. Note that the initial sliding of struts in the 3D re‐entrant unit cells is due to the stress imbalance caused by the slight deviation in the contact point between horizontal struts of adjacent unit cells interlocked in the structured fabric. The micro‐scale layer‐by‐layer powder spreading of the MJF printing process is horizontal, while the struts of unit cells have a re‐entrant point. Therefore, micro‐scale steps are inevitably induced on the surface of the horizontal struts, which increase the surface roughness of the struts and the friction between adjacent unit cells, thereby causing slight deviations in the contact point between horizontal struts of adjacent unit cells. If the surface of the struts is sufficiently smooth, the horizontal struts on the top surface of the unit cell should be in contact with the re‐entrant point of the downward‐sloping struts of the adjacent unit cells. As a result, distortion‐induced deformation occurs in the structured fabric, and plastic collapse at the re‐entrant point (labeled by the green circle) is avoided.

Therefore, it can be inferred that due to the combined design of 3D re‐entrant unit cells and their novel topological interlocking, the interaction between the horizontal struts in adjacent unit cells and the intruding vertical struts synergistically contribute to the high yield strength and energy absorption of the dual‐faced structured fabric. Furthermore, the distortion of the unit cells under large deformation prevents catastrophic failure of the structured fabric and accommodates large reversible strain, resulting in minimized damage and good deformation recoverability. These phenomena can be further observed in Movie S2, Supporting Information. It is worth noting that changing the fabric material to elastomers could lead to better performance in terms of recoverability. However, the use of elastomers may come at a cost because the synergetic strengthening effect of the 3D re‐entrant unit cells and their novel topological interlocking can be seriously weakened by such materials.

The dual‐faced feature of the structured fabric gives rise to a different bending modulus depending on whether the top or bottom surface is loaded (Figure 3d). The bending modulus of the structured fabric can be enhanced by changing the loading surface (Figure 2d) as well as controlling the vacuum confining pressure, thus expanding the tunable range of the bending modulus. Therefore, a compelling understanding of the underlying deformation mechanism and its link to the dual‐faced feature is critical.

Due to the unique topological interlocking, at the linear elastic stage (small displacement), only the upper part of the structured fabric is subjected to compressive stress, while the central and lower parts remain stress‐free, and only the enveloping film that adheres to the lower surface of the structured fabric is subjected to tensile stress (Section S3, Supporting Information). In addition, the upper part of the structured fabric can be divided into two regions depending on how the unit cells are in contact with each other, that is, the plane contact region and point contact region (Figure 3e). For any infinitesimal segment in the structured fabric, the bending moment can be obtained by the summation of the contributions to the bending moment from the plane contact *M*_1_, point contact *M*_2_, and enveloping film *M*_3_. The bending moment for loading the bottom surface *M* ^B^ and that for the top surface *M* ^T^, are derived in detail using the moment–stress relations and constitutive equations provided in Section S3, Supporting Information.

The effective Young's modulus of every region is affected by how the unit cells are in contact with each other within that region. For the case of loading the bottom surface, as illustrated in the upper right of Figure 3e, the bending moment in an infinitesimal segment is derived as

(1) $$\begin{array}{c} M^{B}=\frac{E_{c1}\int_{\frac{h}{2}-2\delta }^{\frac{h}{2}-\delta }{\left(y_{1}^{B}\right)}^{2}bdy_{1}^{B}+E_{c2}\int_{\frac{h}{2}-\delta }^{\frac{h}{2}}{\left(y_{2}^{B}\right)}^{2}bdy_{2}^{B}+E_{t}\int_{-(\frac{h}{2}+m)}^{-\frac{h}{2}}{\left(y_{3}^{B}\right)}^{2}bdy_{3}^{B}}{\rho } \end{array}$$

where *E*_c1,_ *E*_c2_, and *E*_t_ refer to the Young's moduli of the plane contact region, point contact region, and enveloping film, respectively. $y_{1}^{B}$ is the radial distance of a certain point in the plane contact region relative to the neutral axis, $y_{2}^{B}$ is the radial distance of a certain point in the point contact region relative to the neutral axis, and $y_{3}^{B}$ is the radial distance of a certain point in the enveloping film relative to the neutral axis. *m* is the thickness of the enveloping film, *b* and *h* are the width and height of the structured fabric sample, respectively, and *δ* is the thickness of the regions corresponding to plane contact and point contact. *ρ* is the curvature radius.

Similarly, the bending moment in the case of loading the top surface (the lower right of Figure 3e) is derived as

(2) $$\begin{array}{c} M^{T}=\frac{E_{c1}\int_{\frac{h}{2}-\delta }^{\frac{h}{2}}{\left(y_{1}^{T}\right)}^{2}bdy_{1}^{T}+E_{c2}\int_{\frac{h}{2}-2\delta }^{\frac{h}{2}-\delta }{\left(y_{{}_{2}}^{T}\right)}^{2}bdy_{2}^{T}+E_{t}\int_{-(\frac{h}{2}+m)}^{-\frac{h}{2}}{\left(y_{3}^{T}\right)}^{2}bdy_{3}^{T}}{\rho } \end{array}$$

where $y_{1}^{T}$ is the radial distance of a certain point in the plane contact region relative to the neutral axis, $y_{2}^{T}$ is the radial distance of a certain point in the point contact region relative to the neutral axis, and $y_{3}^{T}$ is the radial distance of a certain point in the enveloping film relative to the neutral axis. *ρ* is the curvature radius, which is the same in both cases of loading the bottom and top surfaces due to the same loading displacement.

The difference in the bending moments is calculated as

(3) $$M^{T}-M^{B}=\frac{\left(E_{c1}-E_{c2}\right)}{\rho }\frac{b}{3}\left(3h\delta^{2}-6\delta^{3}\right)=55.8\frac{\left(E_{c1}-E_{c2}\right)}{\rho }>0$$

where *b* and *h* are about 50 and 13 mm, respectively, and *δ* is approximately equal to the re‐entrant distance, that is, 0.3 mm.

As *E*_c1_ is always greater than *E*_c2_, Equation (3) indicates that the bending moment is larger when the top surface is loaded than when the bottom surface is loaded. The unique topological interlocking of unit cells is responsible for the difference in the bending modulus by introducing plane and point contact regions on the top and bottom surfaces, respectively. Therefore, the advanced mechanical properties of the structured fabric hinges largely on the design of the re‐entrant unit cells and their novel topological interlocking.

### 2.4 Performance Analysis and Evaluation

**Figure 4:**
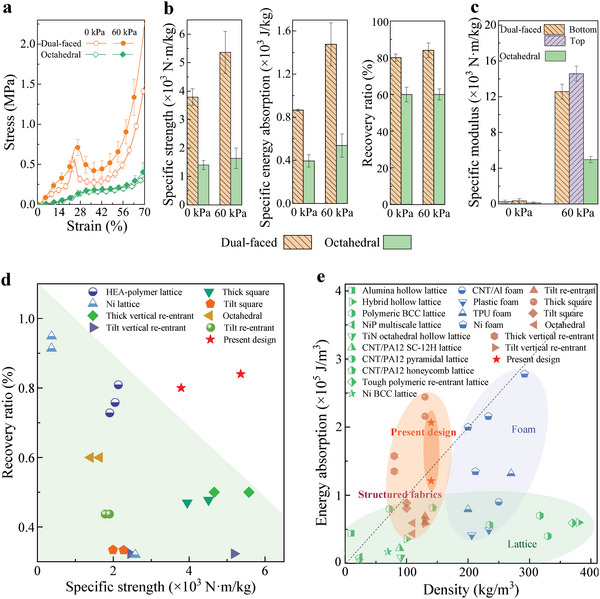
Mechanical properties of the dual‐faced structured fabric compared against those of other energy‐absorbing structures. a) Compressive stress–strain curves of the dual‐faced structured fabric and octahedral structured fabric under different vacuum confining pressures. b) Specific strength, specific energy absorption, and recovery ratios of the two structured fabrics under different vacuum confining pressures. c) Specific bending moduli of the two structured fabrics under the corresponding conditions. Note that the data for the dual‐faced structured fabric under the vacuum confining pressure of 0 kPa in (a–c) is reproduced from Figure 2. d) Recovery ratio versus strength for the dual‐faced structured fabric (present design), other structured fabrics, and lattice structures.^[^ ^19^, ^20^, ^21^ ^]^ e) Ashby map of energy absorption per unit volume versus density, comparing the energy absorption capacity of the designed dual‐faced structured fabric against those of other structured fabrics, foam materials, and lattice structures.^[^ ^8^, ^14^, ^19^, ^20^, ^21^, ^22^, ^23^, ^24^, ^25^, ^26^, ^27^, ^28^, ^29^, ^30^ ^]^ Note that the data provided in (d) and (e) for the different structured fabrics are obtained under vacuum confining pressures of 0 and 60 kPa. Detailed information of the reference data is provided in Section S6, Supporting Information.

To highlight the excellent mechanical properties of the dual‐faced structured fabric, the results from the compression, three‐point bending, and cyclic compression tests were first compared against those of a recently proposed structured fabric.^[^ ^15^ ^]^ The structured fabric considered here for comparison is fabricated using the same technique and material as the designed dual‐faced structured fabric, that is, MJF and PA12. Different from the dual‐faced structured fabric, the fabric in^[^ ^15^ ^]^ consists of hollow octahedral unit cells and has no dual‐faced feature, that is, it has symmetric top and bottom surfaces (hereafter referred to as the octahedral structured fabric). **Figure** **4**a displays the compression stress–strain curves of the two structured fabrics. The octahedral structured fabric undergoes plastic yielding at a relatively low stress level and continues to deform under approximately constant stress until the final densification stage. Additionally, during the entire deformation process, the stress within the dual‐faced structured fabric is always greater, indicating its superior response to compressive loading.

The specific strength and specific energy absorption of the two structured fabrics were calculated (see Experimental Section) and are shown in Figure 4b. Both with and without vacuum confining pressure, the specific strength and specific energy absorption of the dual‐faced structured fabric are evidently higher than those of the octahedral structured fabric. Under a vacuum pressure of 60 kPa, the specific strength and specific energy absorption are up to 3.4 and 2.8 times higher, respectively. The recovery ratio of the octahedral structured fabric was calculated to be about 60% after a compression strain of 70% was applied under vacuum pressures of 0 and 60 kPa, which is smaller than that of the dual‐faced structured fabric (≈80%). The result can be explained by analyzing the deformation mechanism of the octahedral structured fabric. The struts in the octahedral unit cells undergo elastic buckling, plastic collapse, and brittle fracture in succession as the deformation progresses. Consequently, the octahedral structured fabric experiences severe permanent damage and displays poor deformation recoverability (see Section S4 and Movie S3, Supporting Information).

In addition, the hysteresis loop continuously shrinks during the entire cyclic compression test for the octahedral structured fabric (Section S2, Supporting Information). The phenomenon is not only related to the accumulation of plastic deformation, but also to the gradual failure of the vacuum confining pressure as the film was ruptured by the rugged surface of the structure during prolonged cycling. A detailed analysis is given in Section S2, Supporting Information. Moreover, the energy loss coefficient of the octahedral structured fabric is about 44%, which is 16% higher than that of the dual‐faced structured fabric, indicating the relatively poor durability of the former.^[^ ^31^, ^32^ ^]^

The two structured fabrics exhibit similar bending responses, including an initial linear elastic behavior and a subsequent nonlinear response (see Section S5, Supporting Information). However, the specific bending modulus of the dual‐faced structured fabric is found to be up to 2.9 times larger than that of the octahedral structured fabric under a vacuum confining pressure of 60 kPa (Figure 4c).

To further evaluate the performance of our structured fabric, five other structured fabrics constructed using different unit cells or topological interlocking were designed and manufactured from PA12 using the MJF technique (Table S1, Supporting Information). The mechanical properties of the dual‐faced structured fabric were compared against those of the octahedral structured fabric, the five manufactured structured fabrics, and other lattice structures and foam materials. Figure 4d shows that the dual‐faced structured fabric has an outstanding combination of high recovery ratio and high specific strength. The results suggest that the combined design of structural unit cells and their novel topological interlocking provide an efficient route to overcome the strength–recoverability trade‐off for structured fabrics.

In the context of lightweight design, one of the most important features used to evaluate a material's energy absorption capacity is its specific energy absorption, that is, the ratio between its energy absorption per unit volume and density. Hence, the Ashby map of energy absorption per unit volume versus density for the designed structured fabric and other structured fabrics, foam materials, and lattice structures is given in Figure 4e. The energy absorption per unit volume of the dual‐faced structured fabric is about three times larger than that of other structured fabrics with comparable densities. Although the energy absorption of the thick‐square structured fabric is slightly higher than that of the dual‐faced structured fabric, the recovery ratio of the former is significantly lower (Figure 4d). We introduced the scaling law (the dotted line in Figure 4e) for the relationship between the energy absorption and density, and found that the dual‐faced structured fabric has a larger scaling exponent than most of the recently reported energy‐absorbing foam materials and lattices. This observation indicates that the dual‐faced structured fabric has higher energy absorption than other structured fabrics, foam materials, and lattices with comparable densities.

### 2.5 Applications in Shape‐Adaptive Protection

**Figure 5:**
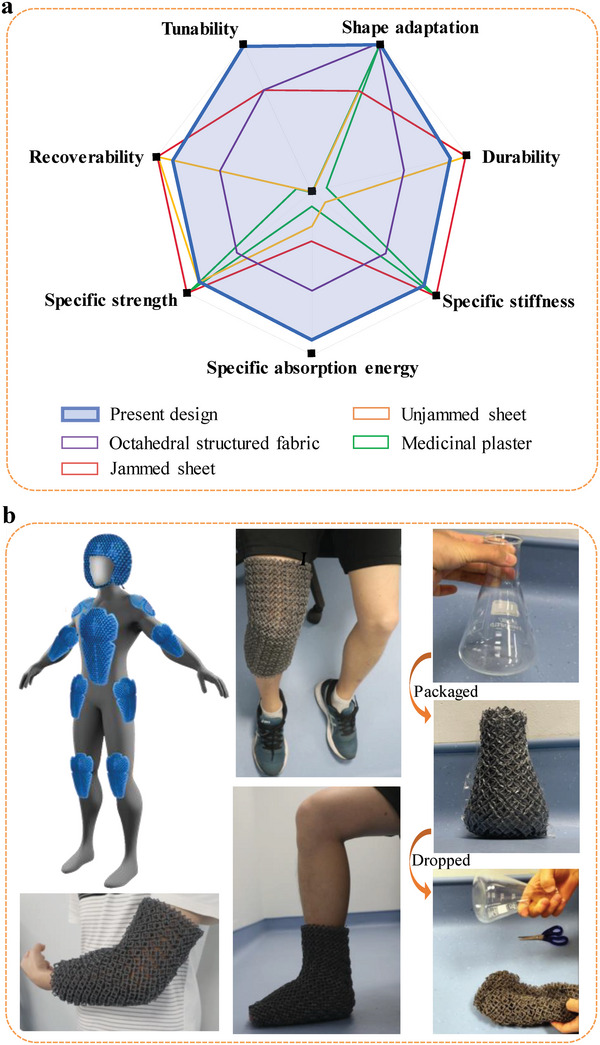
Feature comparison and applications. a) Radar chart qualitatively showing a feature comparison between the dual‐faced structured fabric (present design) and other reported materials for wearable applications, including the jammed octahedral structured fabric,^[^ ^15^ ^]^ jammed and unjammed sheets,^[^ ^30^ ^]^ and medicinal plaster.^[^ ^28^ ^]^ b) Proposed applications of the dual‐faced structured fabric in shape‐adaptive protection, including lightweight body armor, medical support, and protective packaging.

The investigated properties of the dual‐faced structured fabric demonstrate promising potential for the material to be used in wearable protection and medical support. **Figure** **5**a provides a radar chart for the overall performance evaluation of the dual‐faced structured fabric and previously reported materials for wearable applications.^[^ ^15^, ^28^, ^30^ ^]^ Herein, seven performance metrics including the specific energy absorption, specific bending stiffness, specific strength, recoverability, durability, shape adaptation, and tunability of the mechanical properties were selected for the assessment. The benchmarks for comparison are detailed in Section S7, Supporting Information. As shown in Figure 5a, the dual‐faced structured fabric is the only one capable of balancing high performance in all seven criteria. Notably, the dual‐faced structured fabric is found to compare favorably in terms of its performance against a wide range of materials proposed for wearable applications. The excellent mechanical and structural properties would extend the application prospects of the structured fabric from medical support, exoskeletons, and robotics to the protection of the human body and precision devices.

For the octahedral structured fabric, despite the vacuum confining pressure allowing for a tunable bending modulus, it does not possess the strengthening mechanism required to support high specific strength and specific energy absorption. It also lacks the dual‐faced feature which broadens the tunable range of the bending modulus, and the distortion‐induced deformation mechanism which promotes good recoverability. For the jammed or unjammed sheets, their poor shape adaptivity restricts the material from accessing complex shapes with compound curvature. Furthermore, the tunable bending stiffness and superior energy absorption of the dual‐faced structured fabric can compensate for the limitations posed by medicinal plaster, making it a highly suitable candidate material for the medical industry. For example, the inflexibility of medicinal plaster results in stiffness of the injured limbs, while its heavy mass limits the patients’ mobility.^[^ ^28^ ^]^ The lightweight structured fabric can not only fixate the injured limb by applying vacuum confining pressure around its boundary, but also allow for rehabilitative movement by releasing the confining pressure and reducing its bending stiffness (see Movie S4, Supporting Information). In addition, the superior energy absorption capacity can protect the injured limbs and avoid causing secondary damage during the medical fixation period, thereby promoting better recovery and improving the treatment efficiency. Besides, the dual‐faced structured fabric can be reusable, which would save resources and medical costs, and its relatively smooth surface compared to other structured fabrics^[^ ^15^ ^]^ will not only enhance the comfort of the patient, but also extend its applications to wearable exoskeletons and lightweight body armor.

For the dual‐faced structured fabric, the large specific bending stiffness and high specific yield strength and specific energy absorption under vacuum confining pressure extend its application from providing unobtrusive physical support for users with joint instabilities to serving as precision component packaging. The simplest form of shape locking can be achieved by manually bending the unjammed structured fabric into a desired shape and then jamming the structure under a vacuum confining pressure. For example, the designed structured fabric was used to encase glassware by first shaping it to the curved surface of the glass (right column in Figure 5b). By applying a vacuum confining pressure of 60 kPa, the structured fabric was jammed and able to protect the glassware from breaking after 20 drops from a height of 1 m whereas the unprotected glassware broke after one drop from 0.4 m (see Movie S5, Supporting Information). Note that the structured fabric can be restored back to its original form and reused after releasing the vacuum confining pressure.

We reiterate that the dual‐faced structured fabric possesses excellent energy absorption capacity, and believe that this work can contribute to the development of advanced rehabilitative garments for injuries with protection against secondary damage and lightweight smart clothing with protective capabilities. The design further enables individuals, such as astronauts or field rescuers, to rapidly create tools and structures of any shape and adapt their performance on‐demand in resource‐scarce conditions. Despite the dual‐faced structured fabric being fabricated from PA12 by the MJF technique in the present work, the rapid advancement of additive manufacturing enables the structure to be processed by other materials and printing techniques depending on the application requirements. As an example, for ultra‐high strength, high temperature, and/or shape‐adaptive protection applications, the structured fabric can be printed using a superalloy.^[^ ^33^ ^]^ These capabilities would broaden the application of structured fabrics toward shape‐adaptive protection such as medical support and precision component packaging, as well as military and aerospace safety equipment.

## 3 Conclusion

Enabled by additive manufacturing, we designed a dual‐faced structured fabric consisting of 3D re‐entrant unit cells with a novel topological interlocking to achieve superior mechanical properties and shape adaptation. The dual‐faced structured fabric exhibits excellent specific energy absorption, outstanding specific strength, and high recovery ratio (≈80% after a compression strain of 70% is applied), hence overcoming the strength–recoverability trade‐off, ascribed to the distortion‐induced deformation. Additionally, the specific bending modulus can be improved by 67 times when a small vacuum confining pressure of 90 kPa was applied around the boundaries of the dual‐faced structured fabric. The topological interlocking results in different unit cell arrangements along the top and bottom surfaces of the structured fabric, thus contributing to the enhanced mechanical properties, in addition to broadening the tunable range of the bending modulus. The dual‐faced structured fabric outperforms other structured fabrics fabricated using the same material and additive manufacturing technology. For example, it demonstrates a 3.4 times higher specific strength, a 2.8 times higher specific energy absorption, and a 2.9 times higher specific bending modulus than the octahedral structured fabric, when the same vacuum confining pressure of 60 kPa was applied. Due to its unique combination of mechanical and structural properties, the dual‐faced structured fabric complements existing materials suggested for wearable applications and presents itself as a viable option for lightweight shape‐adaptive protection and multifunctional medical support.

## 4 Experimental Section

### Materials and Manufacturing

The structured fabrics were printed by an HP Jet Fusion 3D 5200 printer (HP Inc., USA) using PA12 powder with an average size of ≈66.2 µm and a peak melting temperature of 188.4 °C.^[^ ^32^ ^]^ The “Balanced” print mode was selected for the printing of structured fabrics using a recommended virgin/used powder mixture ratio of 20:80. The thickness of the printing layer was 80 µm. After fabrication, all structures were subjected to a sand blasting treatment with glass beads (average size of ≈90 µm) at pressure of ∼0.4 MPa. Finally, the post processed parts were encased within an envelope composed of a thermoplastic polyurethane film with a thickness of 0.1 mm. A vacuum confining pressure was then applied to jam the structured fabric.

### Quasi‐Static and Cyclic Compression Tests

The quasi‐static compression and cyclic compression tests were performed on the confined fabric by a universal testing machine (Shimadzu AGX, Japan). For the quasi‐static compression test, a constant displacement rate of 0.01 m s^−1^ was applied; while for the quasi‐static cyclic compression test, the cyclic loading was repeated 30 times at a strain amplitude of 25% and a constant displacement rate of 0.1 mm s^−1^. To quantitatively evaluate the mechanical properties of the structured fabric, the yield strength, recovery ratio, energy absorption, energy absorption efficiency, and energy loss coefficient were investigated. The yield strength was extracted from the compression stress–strain curves. To compare between the different structured fabrics, the yield strength and energy absorption were normalized by calculating the specific strength and specific energy absorption, which were defined as the ratios of the yield strength and energy absorption to the apparent density of the structured fabric sample, respectively. The recoverability was evaluated by the recovery ratio, which was defined as the ratio of the recovered strain to the total applied strain.

The energy absorbed per unit volume under compressive loading *W*_loading_ is represented by the area under the compression stress–strain curve and is given by

(4) $$W_{\mathrm{loading}}=\int_{0}^{\varepsilon }\sigma \left(\varepsilon \right)d\varepsilon$$

where *σ* and *ε* refer to the stress and strain, respectively.

The specific energy absorption *ϕ* can be calculated as

(5) $$\phi =\frac{W_{\mathrm{loading}}}{\rho_{a}}=\frac{\int_{0}^{\varepsilon }\sigma (\varepsilon )d\varepsilon }{\rho_{a}}$$

where *ρ*_a_ refers to the apparent density of the structured fabric sample, defined as the ratio of the mass to the volume that the printed sample occupies.

The energy absorption efficiency *µ* is defined as the ratio of the energy absorbed by the structure to the energy absorbed by an ideal energy absorber when both produce the same peak stress *σ*_p_, and is given by

(6) $$\mu =\frac{\int_{0}^{\varepsilon }\sigma \left(\varepsilon \right)d\varepsilon }{\sigma_{p}}$$

The energy loss coefficient *η* for cyclic compression is evaluated by

(7) $$\eta =\frac{W_{\mathrm{loading}}-W_{\mathrm{unloading}}}{W_{\mathrm{loading}}}\times 100\%$$

where *W*_loading_ and *W*_unloading_ are the areas under the loading and unloading paths for the compressive stress–strain curve of every cycle, respectively.^[^ ^32^, ^34^ ^]^

### Bending Test

The static three‐point bending test was performed at room temperature using the Shimadzu AGX testing machine with a crosshead displacement speed of 10 mm min^−1^. The distance between the outer rollers was 80 mm. The length *l*, width *b*, and thickness *h* of the fabricated samples under different vacuum confining pressures were all measured before the three‐point bending test. The two edges of the samples were supported, and their top and bottom surface centers were perpendicularly loaded by a line‐shaped indenter. Each test was performed on three specimens to obtain a mean value. The corresponding apparent bending modulus *E* is calculated based on the sample dimensions and the slope of the elastic regime according to

(8) $$E=\frac{F_{e}l^{3}}{4d_{e}bh^{3}}$$

where *F*_e_ is the measured load and *d*_e_ is the corresponding displacement.

To compare the bending properties of the different structured fabrics, the specific modulus *φ* is calculated by normalizing the bending modulus as

(9) $$\varphi =\frac{E}{\rho_{a}}=\frac{F_{e}l^{3}}{4d_{e}bh^{3}\rho_{a}}$$

## Conflict of Interest

The authors declare no conflict of interest.

## Supporting information

Supporting Information

Supplemental Movie 1

Supplemental Movie 2

Supplemental Movie 3

Supplemental Movie 4

Supplemental Movie 5

## Data Availability

The data that support the findings of this study are available in the supplementary material of this article.
