# Supplementary material for: Additively Manufactured Dual‐Faced Structured Fabric for Shape‐Adaptive Protection
Source: Adv Sci (Weinh). 2023 May 10;10(21):2301567. doi: 10.1002/advs.202301567 (PMC10375195; doi:10.1002/advs.202301567)
Supplement: Supplementary file 1 — Supporting Information [file ADVS-10-2301567-s003.pdf]

ADVANCED SCIENCE
Open Access

Supporting Information

for *Adv. Sci.*, DOI 10.1002/advs.202301567

Additively Manufactured Dual-Faced Structured Fabric for Shape-Adaptive Protection

Yuanyuan Tian, Kaijuan Chen, Han Zheng, Devesh R. Kripalani, Zhuohong Zeng, Asker Jarlöv, Jiayao Chen, Lichun Bai, Adrian Ong, Hejun Du, Guozheng Kang, Qihong Fang, Lihua Zhao, H. Jerry Qi, Yifan Wang and Kun Zhou**

Supporting Information

Additively Manufactured Dual-faced Structured Fabric for Shape-adaptive Protection

Yuanyuan Tian #, Kaijuan Chen #, Han Zheng, Devesh R. Kripalani, Zhuohong Zeng, Asker Jarlöv, Jiayao Chen, Lichun Bai, Adrian Ong, Hejun Du, Guozheng Kang, Qihong Fang, Lihua Zhao, H. Jerry Qi, Yifan Wang, Kun Zhou**

Section 1. Monotonic compression responses of the dual-faced and octahedral structured fabrics

Figures S1 and S2 show the compression stress–strain curves and the energy absorption efficiency–strain curves of the dual-faced and octahedral structured fabrics. The critical strain at the onset of densification ε_D and the corresponding stress σ_D is obtained at the highest energy absorption efficiency. Here, the amount of energy absorbed is obtained by calculating the area under the compression stress–strain curve in the strain ranges from 0 to ε_D [1]. The highest energy absorption efficiency is about 30% for both the dual-faced and octahedral structured fabrics under various vacuum confining pressures.

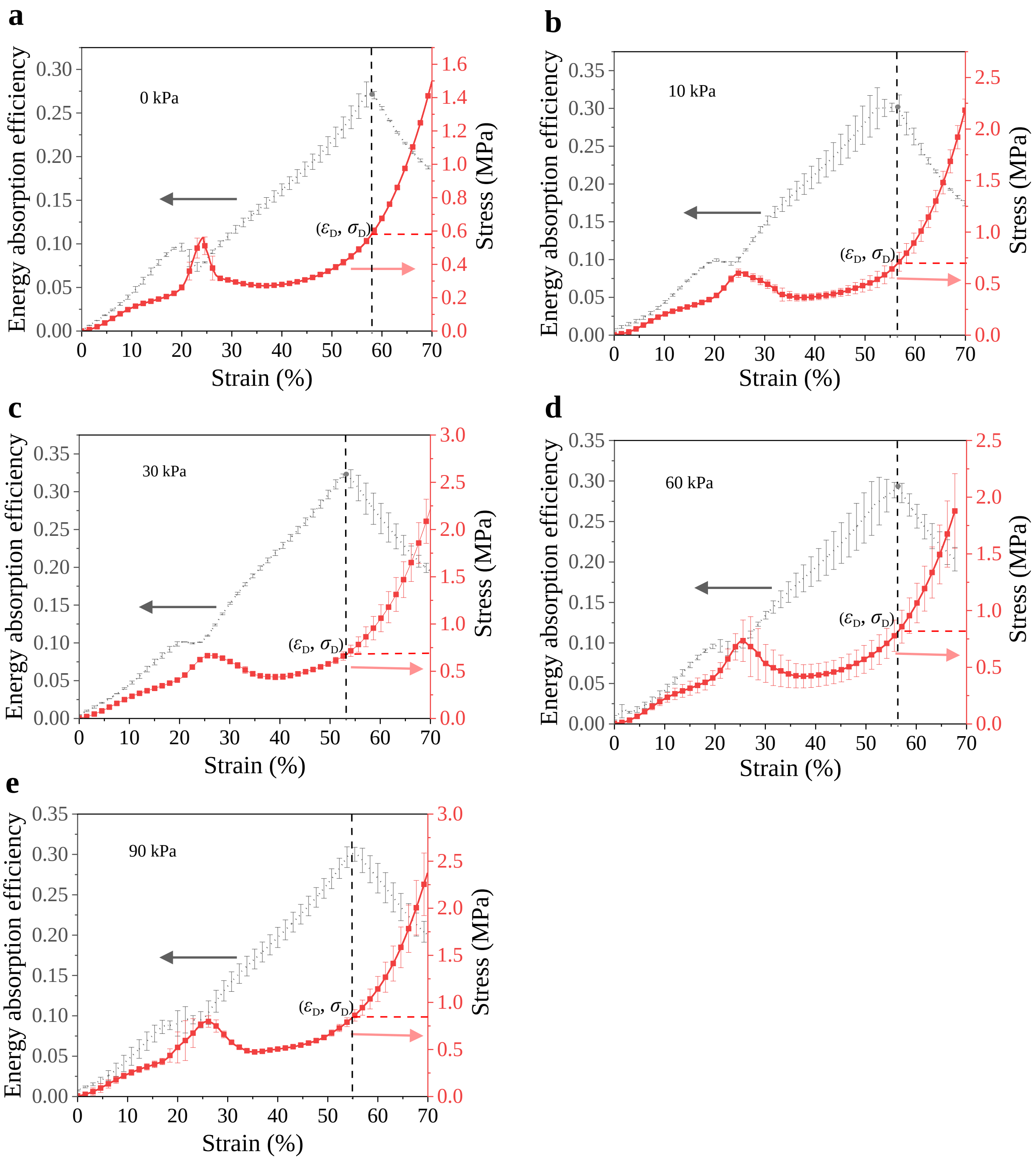

Figure S1. Compression stress–strain and energy absorption efficiency–strain curves of the dual-faced structured fabric under different vacuum confining pressures of **a,** 0 kPa, **b,** 10 kPa, **c,** 30 kPa, **d,** 60 kPa, and **e,** 90 kPa.

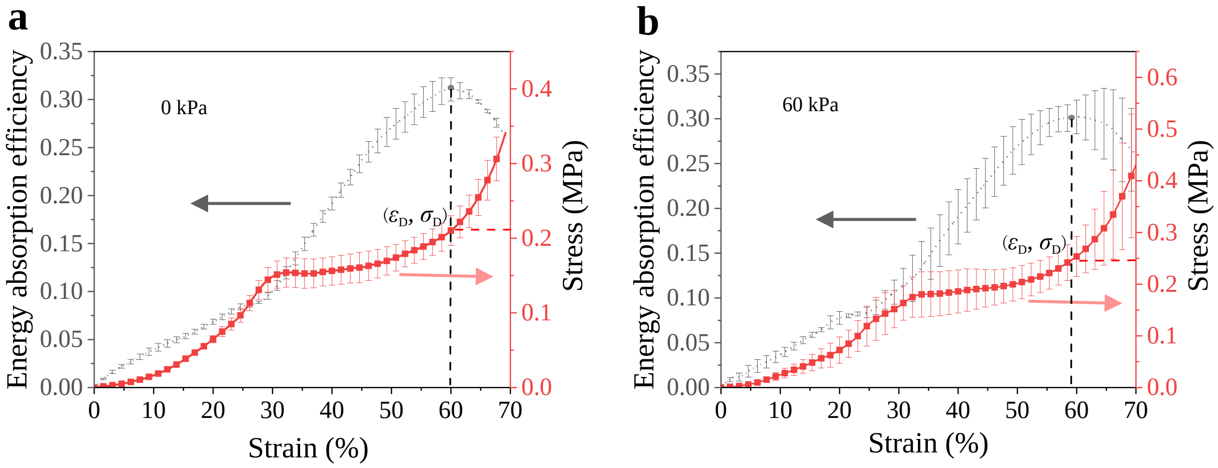

Figure S2. Compression stress–strain and energy absorption efficiency–strain curves of the octahedral structured fabric under vacuum confining pressures of **a**, 0 kPa and **b**, 60 kPa.

Section 2. Cyclic compression of the dual-faced and octahedral structured fabrics

Cyclic compression tests were performed to comparatively investigate the durability of the dual-faced and octahedral structured fabrics. **Figures S3a and S3b** show the cyclic compression stress–strain curves of the two structured fabrics under a vacuum confining pressure of 60 kPa. A compression strain of 25% was applied for each compressive cycle. Both structured fabrics exhibited hysteresis during their deformation which might be attributed to the friction between the struts of adjacent unit cells, as well as the accumulated plastic deformation of the base material, especially at the structural joints.

For the dual-faced structured fabric, the hysteresis loop shrinks during the first two cycles and remains almost unchanged from the third cycle onwards, indicating that the cyclic response stabilizes after approximately three cycles (**Figure S3a**). This result reveals that the accumulation of plastic deformation occurs mainly in the first two cycles and after that, mainly elastic deformation occurs. For the octahedral structured fabric, however, the hysteresis loop keeps shrinking during the entire cyclic compression test, which is not only

related to the accumulation of plastic deformation, but also to the gradual failure of vacuum confining pressure with the increase in the number of cycles. For the dual-faced structured fabric, the vacuum confining pressure is preserved after 30 compression cycles (**Figure S3c**). The failure of the vacuum confining pressure is attributed to the puncturing of the enveloping film by the sharp corners of the octahedral structured fabric (**Figure S3d**).

In addition, the energy loss coefficients of the dual-faced and octahedral structured fabrics were stable at about 38% and 44%, respectively, during cyclic compression (see Methods). Moreover, the energy loss coefficient of the dual-faced structured fabric is as low as that of three-dimensional (3D) thermoplastic polyurethane (TPU) lattice foam [2]. This finding shows that the dual-faced structured fabric has excellent energy restoration and long-lasting performance under long-term cyclic loading.

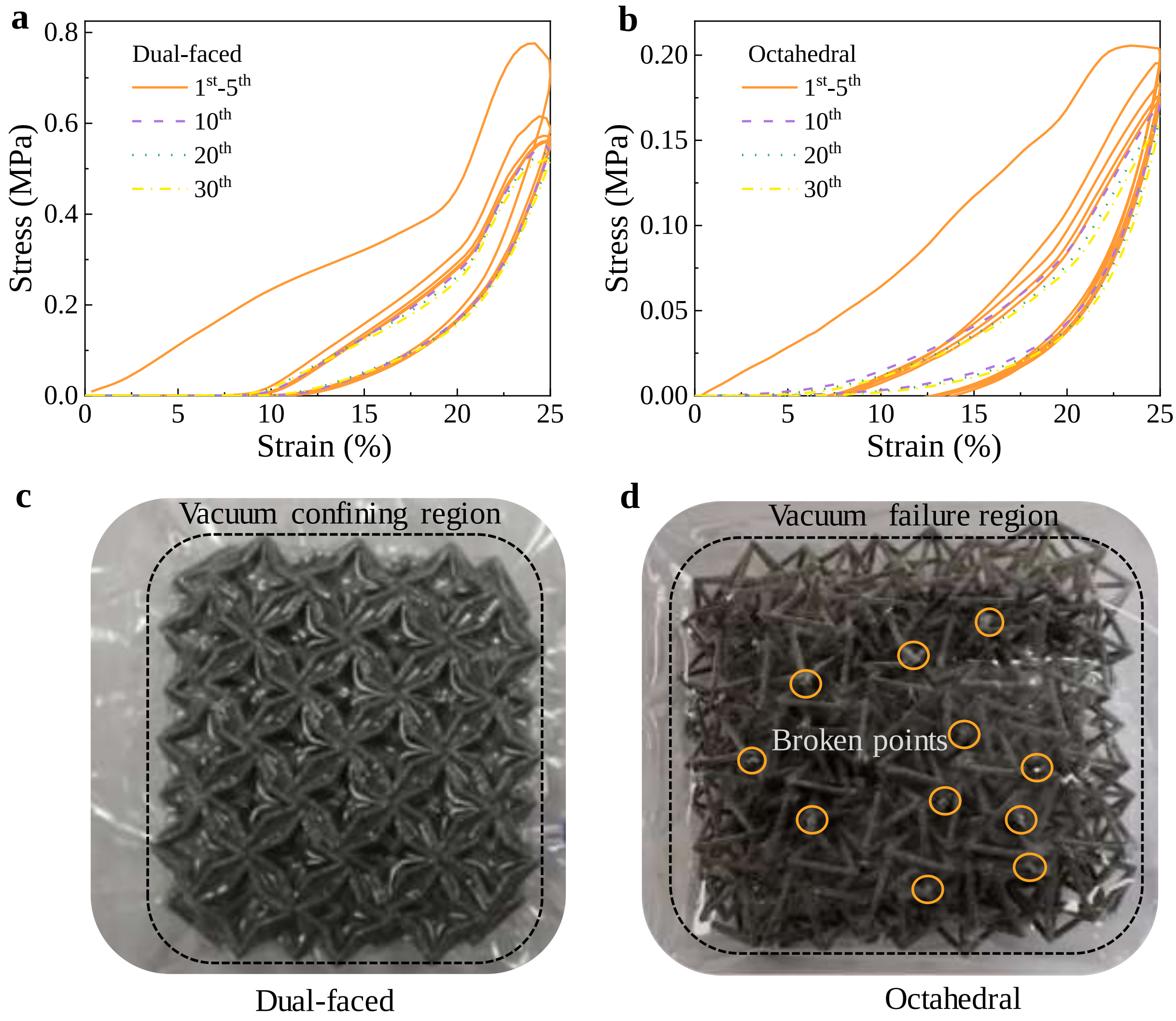

Figure S3. a, b, Cyclic compression test results of the dual-faced and octahedral structured fabrics. **c**, **d,** Surface morphologies of the dual-faced and octahedral structured fabrics after 30 compression cycles.

Section 3. Mechanical response and analytical modelling of bending deformation of the dual-faced structured fabric

The upper part of the dual-faced structured fabric consists of the plane contact region and point contact region (**Figure 3e** in the manuscript). At the linear elastic stage (small displacement), the upper part of the structured fabric is subjected to compressive stress due to the interaction between unit cells induced by plane contact and point contact ("I" of **Figure**

S4). However, the central part is close to the neutral axis of the structured fabric, thus remaining stress-free. As for the lower part, since the structured fabric is a chain mail structure consisting of three-dimensional re-entrant unit cells, they are not in contact across the lower part for small displacement, as shown in “II” of **Figure S4**. The interlocking between the adjacent unit cells will only be evident when the structured fabric experiences large deformation. Therefore, the lower part is stress-free at the linear elastic stage (small displacement) and may be subjected to tensile stress only under large deformation (large displacement).

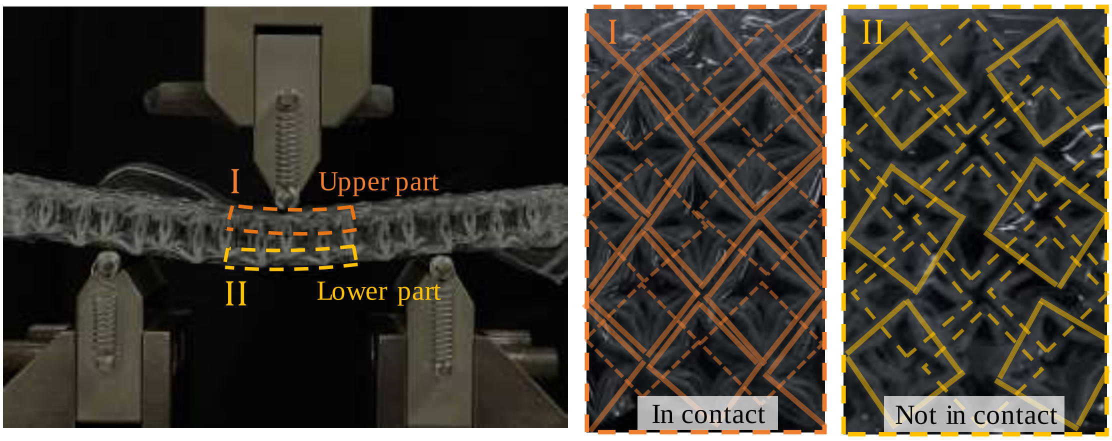

Figure S4. Schematic diagram illustrating the mechanical response of the dual-faced structured fabric during bending deformation with small displacement. The regions consisting of unit cells that are in contact and not in contact with one another are denoted as "I" and "II", respectively.

In addition, the bending tests of single-faced structures were performed to show that the top and bottom surfaces interact with each other during bending deformation. **Figures S5a** and **S5b** show the top views of the two single-faced structures, and the bending test results are given in **Figure S5c**. The specific bending modulus of the single-faced structure with a surface similar to the bottom surface of the dual-faced structured fabric is 7.27×10^3 N·m/kg, which is smaller than that of the dual-faced structured fabric when the bottom surface is

loaded (12.57 × 10^3 N·m/kg). The specific bending modulus of the single-faced structure with a surface similar to the top surface of the dual-faced structured fabric is 12.95 × 10^3 N·m/kg, which is also smaller than that of the dual-faced structured fabric when the top surface is loaded (14.58 × 10^3 N·m/kg). The results indicate that the bottom and top surfaces of the dual-faced structured fabric interact with each other during bending deformation to enhance the bending modulus.

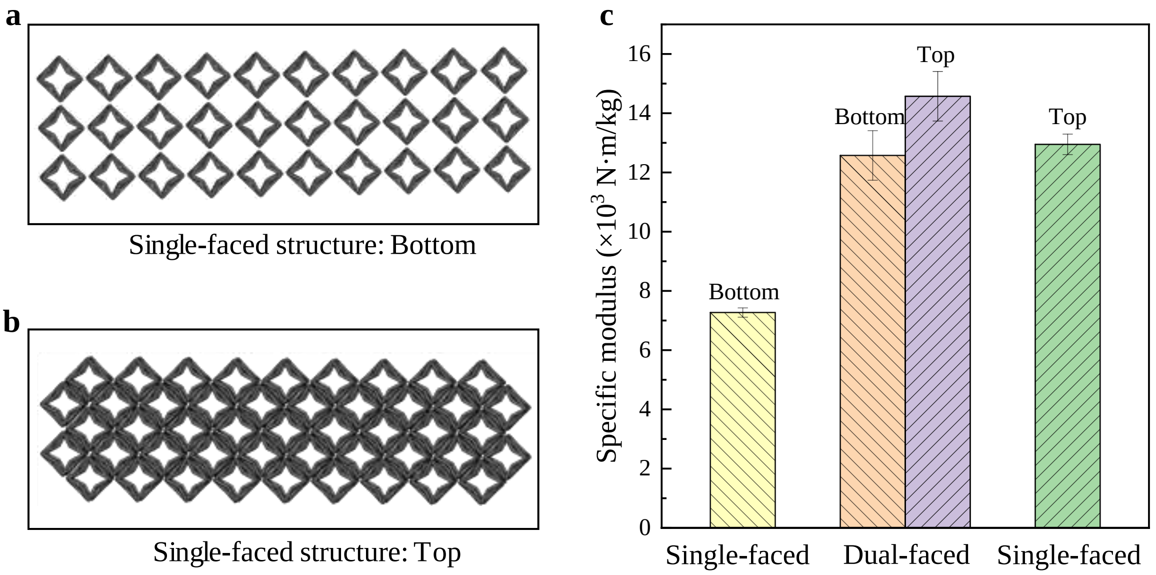

Figure S5. a, b, Top views of the single-faced structure showing the different unit cell arrangements on the surface. **c**, Specific bending modulus of the dual-faced structured fabric and single-faced structures.

Figure S6a shows a schematic illustration of a slender beam under bending deformation. For any infinitesimal segment of the beam (**Figure S6b**), the bending moment M is calculated by

$$M=\int_A y\sigma dA. \tag{1}$$

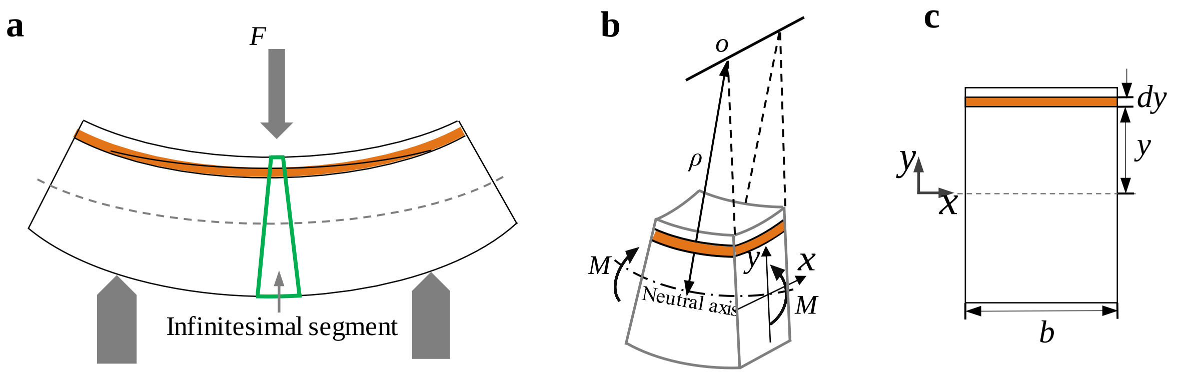

Figure S6. Schematic diagram of a slender beam under bending deformation.

Similar to a slender beam, for any infinitesimal segment in the dual-faced structured fabric, the bending moment can be calculated by Eq. 1. However, due to the topological interlocking, at the linear elastic stage (small displacement), only the upper part of the structured fabric is subjected to compressive stress, while the central and lower parts are stress-free, and only the enveloping film that adheres to the lower surface of the structured fabric is subjected to tensile stress. In addition, the upper part of the structured fabric can be divided into two regions depending on how the unit cells are in contact with each other, *i.e.*, the plane contact region and point contact region (**Figure 3e** in the manuscript). Therefore, the local bending moment in the structured fabric can be obtained by the summation of the contributions to the bending moment from the plane contact region M_1, point contact region M_2, and enveloping film M_3. Note that the contribution from the interaction between the plane contact region and point contact region is ignored since it is similar for the two cases of loading the bottom and top surfaces of the dual-faced structured fabric.

The bending moment in an infinitesimal segment for loading the bottom surface M^{B}, as shown in the upper half of **Figure 3e** in the manuscript is expressed as

$$M^{\mathrm{B}} = \sum_{i=1}^{3} M_i^{\mathrm{B}} . \tag{2}$$

The bending moment contributed by the plane contact region M_1^{B} is calculated by

$$M_1^{\mathrm{B}} = \int_{A_1^{\mathrm{B}}} y_1^{\mathrm{B}} \sigma_{\mathrm{c1}}^{\mathrm{B}} \mathrm{d}A_1^{\mathrm{B}} = \int_{\frac{h}{2}-2\delta}^{\frac{h}{2}-\delta} y_1^{\mathrm{B}} \sigma_{\mathrm{c1}}^{\mathrm{B}} b \mathrm{d}y_1^{\mathrm{B}}, \tag{3}$$

where y_1^{B} is the radial distance of a certain point in the plane contact region relative to the neutral axis. *b* and *h* are the width and height of the infinitesimal segment, and δ is the thickness of the region corresponding to plane contact (upper half of **Figure 3e** in the manuscript).

The effective Young's moduli of the plane contact region, point contact region, and enveloping film are referred to as E_{c1}, E_{c2}, and E_{t}, respectively. The $\sigma_{\mathrm{c1}}^{\mathrm{B}}$ can be calculated by

$$\sigma_{\mathrm{c1}}^{\mathrm{B}} = E_{\mathrm{c1}} \varepsilon_{\mathrm{c1}}^{\mathrm{B}}, \tag{4}$$

where the corresponding compression strain $\varepsilon_{\mathrm{c1}}^{\mathrm{B}}$ can be calculated by

$$\varepsilon_{\mathrm{c1}}^{\mathrm{B}} = \frac{y_1^{\mathrm{B}}}{\rho}, \tag{5}$$

where ρ is the curvature radius of the infinitesimal segment and y_1^{B} is the radial distance of a certain point in the region relative to the neutral axis.

The substitution of Eqs. (4) and (5) into Eq. (3) results in

$$M_1^{\mathrm{B}} = \int_{\frac{h}{2}-2\delta}^{\frac{h}{2}-\delta} y_1^{\mathrm{B}} E_{\mathrm{c1}} \frac{y_1^{\mathrm{B}}}{\rho} b \mathrm{d}y_1^{\mathrm{B}} = \frac{E_{\mathrm{c1}}}{\rho} \int_{\frac{h}{2}-2\delta}^{\frac{h}{2}-\delta} \left(y_1^{\mathrm{B}}\right)^2 b \mathrm{d}y_1^{\mathrm{B}}. \tag{6}$$

The second moment of area for the plane contact region $I_{\mathrm{z1}}^{\mathrm{B}}$ is expressed as

$$I_{\mathrm{z1}}^{\mathrm{B}} = \int_{\frac{h}{2}-2\delta}^{\frac{h}{2}-\delta} \left(y_1^{\mathrm{B}}\right)^2 b \mathrm{d}y_1^{\mathrm{B}}. \tag{7}$$

Thus, Eq. (6) can be simplified to

$$M_1^{\mathrm{B}} = \frac{E_{\mathrm{t1}} I_{\mathrm{z1}}^{\mathrm{B}}}{\rho}. \tag{8}$$

Similarly, the bending moment contributed by the point contact region can be expressed as

$$M_2^{\mathrm{B}} = \frac{E_{\mathrm{t2}} I_{\mathrm{z2}}^{\mathrm{B}}}{\rho}, \tag{9}$$

where $I_{\mathrm{z2}}^{\mathrm{B}}$ is the second moment of area of the point contact region relative to the neutral axis, which can be expressed as

$$I_{\mathrm{z2}}^{\mathrm{B}} = \int_{\frac{h}{2}-\delta}^{\frac{h}{2}} \left(y_2^{\mathrm{B}}\right)^2 b \, \mathrm{d} y_2^{\mathrm{B}}. \tag{10}$$

where y_2^{B} is the radial distance of a certain point in the point contact region relative to the neutral axis.

The bending moment contributed by the enveloping film can be expressed as

$$M_3^{\mathrm{B}} = \frac{E_{\mathrm{t3}} I_{\mathrm{z3}}^{\mathrm{B}}}{\rho}, \tag{11}$$

where $I_{\mathrm{z3}}^{\mathrm{B}}$ is the second moment of area of the enveloping film relative to the neutral axis, which is expressed as

$$I_{\mathrm{z3}}^{\mathrm{B}} = \int_{-(\frac{h}{2}+m)}^{-\frac{h}{2}} \left(y_3^{\mathrm{B}}\right)^2 b \, \mathrm{d} y_3^{\mathrm{B}}, \tag{12}$$

where y_3^{B} is the radial distance of a certain point in the enveloping film relative to the neutral axis and *m* is the thickness of the enveloping film.

By substituting Eqs. (8), (9), and (11) into Eq. (2), the bending moment acting on an infinitesimal segment in the case of loading the bottom surface is derived as

$$M^{\mathrm{B}}=\frac{E_{\mathrm{c}1}I_{\mathrm{z}1}^{\mathrm{B}}+E_{\mathrm{c}2}I_{\mathrm{z}2}^{\mathrm{B}}+E_{\mathrm{t}}I_{\mathrm{z}3}^{\mathrm{B}}}{\rho}. \tag{13}$$

Similarly, for the case of loading the top surface, as shown in the lower half of **Figure 3e** in the manuscript, the bending moment in an infinitesimal segment is given by

$$M^{\mathrm{T}}=\sum_{i=1}^{3}M_i^{\mathrm{T}}, \tag{14}$$

where M_1^{T} can be expressed as

$$M_1^{\mathrm{T}}=\frac{E_{\mathrm{c}1}I_{\mathrm{z}1}^{\mathrm{T}}}{\rho}, \tag{15}$$

where $I_{\mathrm{z}1}^{\mathrm{T}}$ is the second moment of area of the region relative to the neutral axis. $I_{\mathrm{z}1}^{\mathrm{T}}$ can be expressed as

$$I_{\mathrm{z}1}^{\mathrm{T}}=\int_{\frac{h}{2}-\delta}^{\frac{h}{2}}\left(y_1^{\mathrm{T}}\right)^2 b\,\mathrm{d}y_1^{\mathrm{T}}, \tag{16}$$

where y_1^{T} is the radial distance of a certain point in the plane contact region relative to the neutral axis.

The bending moment contributed by the point contact region can be expressed as

$$M_2^{\mathrm{T}}=\frac{E_{\mathrm{c}2}I_{\mathrm{z}2}^{\mathrm{T}}}{\rho}, \tag{17}$$

where $I_{\mathrm{z}2}^{\mathrm{T}}$ is the second moment of area of the point contact region relative to the neutral axis, which is expressed as

$$I_{\mathrm{z}2}^{\mathrm{T}}=\int_{\frac{h}{2}-2\delta}^{\frac{h}{2}-\delta}\left(y_2^{\mathrm{T}}\right)^2 b\,\mathrm{d}y_2^{\mathrm{T}}, \tag{18}$$

where y_2^{T} is the radial distance of a certain point in the point contact region relative to the neutral axis.

The bending moment contributed by the enveloping film can be expressed as

$$M_3^{\mathrm{T}} = \frac{E_{\mathrm{t}} I_{\mathrm{z3}}^{\mathrm{T}}}{\rho}, \tag{19}$$

where $I_{\mathrm{z3}}^{\mathrm{T}}$ is the second moment of area of the enveloping film relative to the neutral axis. $I_{\mathrm{z3}}^{\mathrm{T}}$ can be expressed as

$$I_{\mathrm{z3}}^{\mathrm{T}} = \int_{-(\frac{h}{2}+m)}^{-\frac{h}{2}} \left(y_3^{\mathrm{T}}\right)^2 b \, \mathrm{d}y_3^{\mathrm{T}}, \tag{20}$$

where y_3^{T} is the radial distance of a certain point in the enveloping film relative to the neutral axis.

By substituting Eqs. (15), (17), and (19) into Eq. (14), the bending moment acting on an infinitesimal segment in the case of loading the top surface is derived as

$$M^{\mathrm{T}} = \frac{E_{\mathrm{t1}} I_{\mathrm{z1}}^{\mathrm{T}} + E_{\mathrm{t2}} I_{\mathrm{z2}}^{\mathrm{T}} + E_{\mathrm{t}} I_{\mathrm{z3}}^{\mathrm{T}}}{\rho}. \tag{21}$$

By combining Eqs. (7), (10), (12), (16), (18), and (20), it is shown that

$$\begin{cases} I_{\mathrm{z1}}^{\mathrm{T}} = I_{\mathrm{z2}}^{\mathrm{B}} \\ I_{\mathrm{z2}}^{\mathrm{T}} = I_{\mathrm{z1}}^{\mathrm{B}} \\ I_{\mathrm{z3}}^{\mathrm{T}} = I_{\mathrm{z3}}^{\mathrm{B}} \end{cases}. \tag{22}$$

Based on Eqs. (13), (21), and (22), the difference in the bending moments between loading the top and bottom surfaces of the dual-faced structured fabric is derived as

$$M^{\mathrm{T}} - M^{\mathrm{B}} = \frac{(E_{\mathrm{t1}} - E_{\mathrm{t2}})(I_{\mathrm{z1}}^{\mathrm{T}} - I_{\mathrm{z2}}^{\mathrm{T}})}{\rho}. \tag{23}$$

By substituting Eqs. (16) and (18) into Eq. (23), and taking *b*, *h*, and δ as 50, 13, and 0.3 mm, respectively, the following result can be obtained:

$$M^{\mathrm{T}} - M^{\mathrm{B}} = \frac{(E_{\mathrm{t1}} - E_{\mathrm{t2}})}{\rho} \frac{b}{3} (3h\delta^2 - 6\delta^3) = \frac{55.8(E_{\mathrm{t1}} - E_{\mathrm{t2}})}{\rho} > 0. \tag{24}$$

Note that the effective Young's modulus of the plane contact region E_{c1} is larger than that of the point contact region E_{c2}. Thus, it is shown that M^{T} is greater than M^{B}.

Section 4. Compression responses of the different structured fabrics

Seven structured fabrics, constructed using different unit cells or topological interlocking, were designed. Their physical features, including the configuration, strut diameter, strut length, and apparent density, are presented in Table S1.

Table S1. Physical features of the dual-faced structured fabric and other comparative structured fabrics.

Structured fabric	Configuration	Strut diameter (mm)	Strut length (mm)	Apparent density (kg/m^3)
Dual-faced	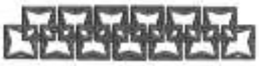	1	10	140
Octahedral	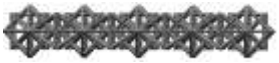	1	10	110
Thick vertical re-entrant	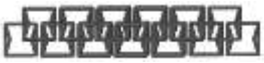	1	10	80
Thick square	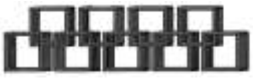	1	10	130
Tilt re-entrant	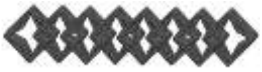	1	10	130
Tilt vertical re-entrant	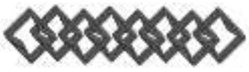	1	10	130
Tilt square	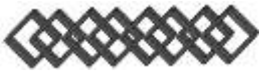	1	10	100

Figure S7 shows the deformed structures of the comparative structured fabrics, namely the octahedral, tilt re-entrant, thick vertical re-entrant, tilt vertical re-entrant, thick square, and tilt square structured fabric, after compression tests in which a strain of 70% was applied. It is

observed that some of the struts in the unit cells underwent fracture and hence, these structures displayed poor deformation recoverability.

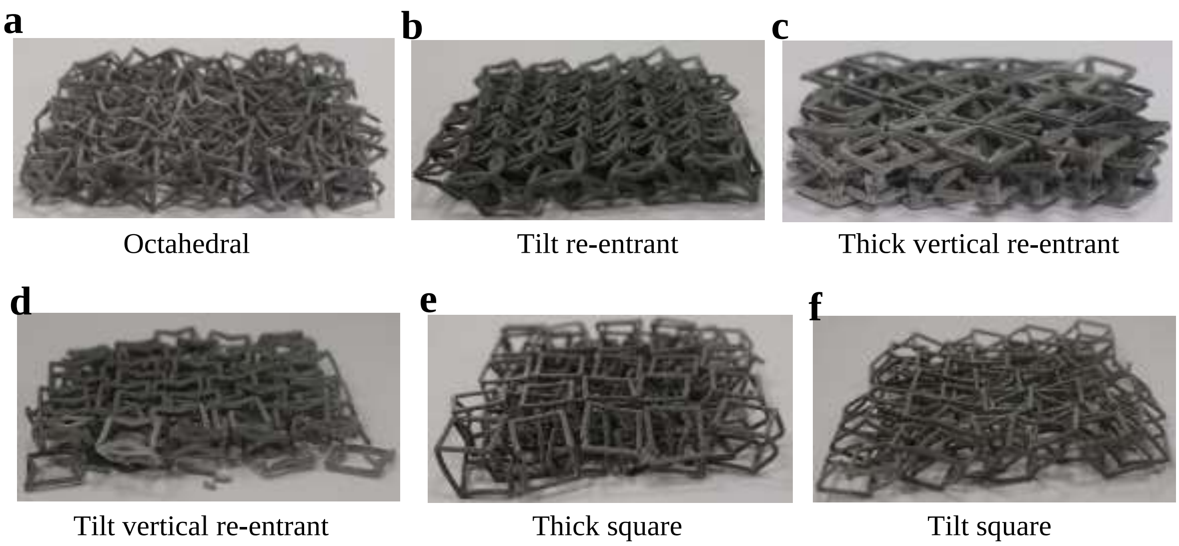

Figure S7. Deformed structured fabrics after the application of 70% compression strain.

Section 5. Bending responses of the dual-faced and octahedral structured fabrics

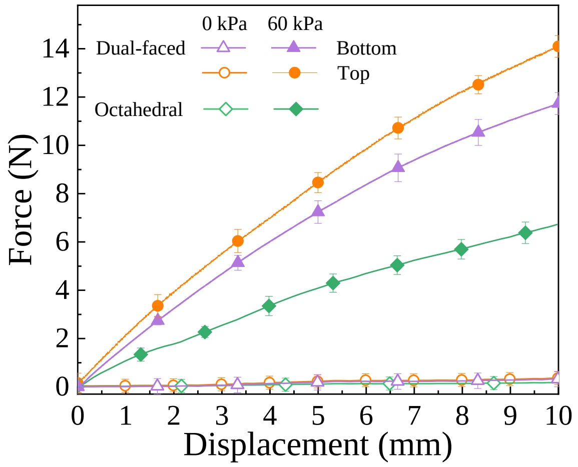

Figure S8. Force–displacement curves during the bending test of the dual-faced and octahedral structured fabrics under vacuum confining pressures of 0 and 60 kPa.

Table S2 gives the difference in the specific bending modulus of the dual-faced structured fabric for the case of loading the bottom and top surfaces under different vacuum confining pressures. The difference decreases when the vacuum confining pressure increases from 10 to 90 kPa. At a lower vacuum confining pressure, the vacuum jamming effect is

weaker. Compared to the frictional drag in the plane contact region on the top surface, the frictional drag in the point contact region on the bottom surface is smaller during bending and can be more easily overcome. However, at a higher vacuum confining pressure, a stronger jamming effect between interlocked unit cells is introduced. Thus, the frictional drag in the point contact region on the bottom surface will increase to be close to that in the plane contact region on the top surface due to a more substantial jamming effect, resulting in minor differences between them. Therefore, the difference in the specific bending modulus of the bottom and top surfaces should be much smaller at a higher vacuum confining pressure of 90 kPa.

Table S2. Difference in the specific bending modulus of the dual-faced structured fabric for the case of loading the bottom and top surfaces under different vacuum confining pressures.

Specific bending modulus ($\times 10^3$ N·m/kg)	Confining pressure (kPa)			
	10	30	60	90
Bottom	8.70	11.29	12.77	17.04
Top	11.64	13.94	14.67	18.10
Difference	2.94	2.65	1.9	1.06

Section 6. Detailed information of the reference data

Table S3. Reference data pertaining to the specific strength and recovery ratio of the different structures.

Structure	Confining pressure (kPa)	Specific strength ($\times 10^3$ N·m/kg)	Recovery ratio (%)	Ref.
Dual-faced structured fabric	0	3.79	80	*
	60	5.36	84	*
Octahedral structured fabric	0	1.4	60	*
	60	1.64	60	*
Thick vertical re-entrant structured fabric	0	4.66	50	*
	60	5.58	50	*

Thick square structured fabric	0	3..95	47	*
	60	4.51	48	*
Tilt re-entrant structured fabric	0	1.79	44	*
	60	1.9	44	*
Tilt vertical re-entrant structured fabric	0	2.46	32	*
	60	5.18	32	*
Tilt square structured fabric	0	1.99	34	*
	60	2.27	33	*
Ni lattice	-	2.58	32	[3, 4]
	-	0.38	95	[3, 4]
	-	0.37	91	[3, 5]
HEA-polymer lattice	-	2.14	81	[3]
	-	2.05	76	[3]
	-	1.91	73	[3]

* Present work

Table S4. Reference data pertaining to the apparent density and energy absorption of the different structures.

Structure	Confining pressure (kPa)	Apparent density (kg/m^3)	Energy absorption ($\times 10^5$ J/m^3)	Ref.
Dual-faced structured fabric	0	140	1.21	*
	60	140	2.07	*
Octahedral structured fabric	0	110	0.43	*
	60	110	0.59	*
Thick vertical re-entrant structured fabric	0	80	1.35	*
	60	80	1.58	*
Thick square structured fabric	0	130	2.16	*
	60	130	2.44	*
Tilt re-entrant structured fabric	0	130	0.60	*
	60	130	0.69	*
Tilt vertical re-entrant structured fabric	0	130	0.58	*
	60	130	0.65	*
Tilt square structured fabric	0	100	0.81	*
	60	100	0.89	*
CNT/Al foam	-	250	0.9	[6]

TPU foam	-	270	0.79	[2]
	-	200	1.32	[2]
Plastic foam	-	206	0.42	[7]
	-	234	0.49	[8]
Ni foam	-	200	2	[7]
	-	233	2.16	[7]
	-	292	2.78	[7, 8]
	-	212	1.35	[7, 8]
Alumina hollow lattice	-	9.6	0.44	[3, 9]
Hybrid hollow lattice	-	100	0.36	[3, 10]
Polymeric BCC lattice	-	330	0.40	[11, 12]
	-	317	0.70	[11, 12]
	-	235	0.56	[11, 12]
NiP multiscale lattice	-	23.7	0.09	[3, 13]
CNT/PA12 SC-12H lattice	-	88.74	0.219	[8]
CNT/PA12 pyramidal lattice	-	72.19	0.80	[8]
CNT/PA12 honeycomb lattice	-	142.54	0.82	[8]
Tough polymeric re-entrant lattice	-	370	0.59	[14]
TiN octahedral hollow lattice	-	90.7	0.09	[3, 15]
Ni BCC lattice	-	69.5	0.17	[3, 4]
	-	23.8	0.02	[3, 4]

* Present work

Section 7. Performance evaluation of materials reported for wearable applications

Table S5 shows a performance comparison of the presently designed dual-faced structure against four other types of materials proposed for wearable applications. As far as possible, scorings are given based on relative performance. When a scoring cannot be given on a quantitative basis, we have attempted to give a reasonable score based on rational interpretation.

Table S5. Comparison of performance for various materials proposed for wearable applications.

Performance metric	Dual-faced structured fabric *	Octahedral structured fabric [16]	Jammed sheet [17]	Unjammed sheet [17]	Medicinal plaster [18]
Specific bending modulus	The calculated value is 14571 N·m/kg at a vacuum confining pressure of 60 kPa. (Score: 9/10)	The calculated value is 4963 N·m/kg at a vacuum confining pressure of 60 kPa. (Score: 6/10)	The calculated value of the jammed sheet is about 1×10^7 N·m/kg at a vacuum confining pressure of 71 kPa. (Score: 10/10)	The unjammed sheet resembles a flat, flexible carpet, enabling it to be easily folded and transported. Compared with the jammed sheet, a very small force (close to zero) is required to achieve the same linear elastic bending deformation. (Score: 1/10)	Medicinal plaster is a hard material. (Score: 10/10)

Specific strength	The calculated value is 5400 N·m/kg at a vacuum confining pressure of 60 kPa. (Score: 9/10)	The calculated value is 1600 N·m/kg at a vacuum confining pressure of 60 kPa. (Score: 6/10)	Compared with the dual-faced and octahedral structured fabrics, the jammed sheet has a higher yield strength when subjected to compression, because it is a dense solid. (Score: 10/10)	The unjammed sheet has a high yield strength when subjected to compression, because it is a dense solid. The yield strength of the unjammed sheet is smaller than that of the jammed sheet. (Score: 9/10)	Medicinal plaster is a hard material. (Score: 10/10)
Specific energy absorption	The calculated value is 1480 J/kg at a vacuum confining pressure of 60 kPa. (Score: 9/10)	The calculated value is 540 J/kg at a vacuum confining pressure of 60 kPa. (Score: 6/10)	The sheet is a thin and dense lamellar material. The jammed sheet is hardened when subjected to a vacuum confining pressure. (Score: 3/10)	The unjammed sheet is a thin and dense lamellar material. (Score: 2/10)	Medicinal plaster is a hard and brittle material. (Score: 1/10)

Durability	The presently designed dual-faced structured fabric quickly achieves stability in its hysteresis response with a small energy loss coefficient of 38%. The membrane remains intact after 30 compression cycles and the jamming effect is well-preserved. (Score: 9/10)	The hysteresis response persists throughout 30 compression cycles and the energy loss coefficient stabilizes at a relatively large value of 44%. In addition, the jamming effect tends to fail easily because the membrane is prone to rupture which is caused by contact with the sharp corners of the octahedral unit cells. (Score: 6/10)	The jammed sheet is a thin and dense lamellar material. It does not break easily and can be used repeatedly. (Score: 10/10)	The unjammed sheet is a thin and dense lamellar material. It does not break easily and can be used repeatedly. (Score: 10/10)	Medicinal plaster is disposable and undergoes brittle fracture easily during use. (Score: 1/10)
Recoverability	The calculated value of the recovery ratio is approximately 80%. (Score: 9/10)	The calculated value of the recovery ratio is approximately 60% with some fractured struts. (Score: 6/10)	The jammed sheet is a thin and dense lamellar material. (Score: 10/10)	The jammed sheet is a thin and dense lamellar material. (Score: 10/10)	The recoverability of medicinal plaster is poor because it is a brittle material. (Score: 1/10)

Shape adaptation	The presently designed dual-faced structured fabric is a chained structure, which has good shape adaptation. (Score: 10/10)	The octahedral structured fabric is a chained structure, which has good shape adaptation. (Score: 10/10)	Compared with chained structures, the sheet has relatively poor shape adaptation. It is challenging for jammed sheet to achieve high material malleability and realize complex shapes with compound curvatures, as the sheet materials are not stretchable or can be only stretched in one direction. (Score: 7/10)	Compared with chained structures, the sheet has poor shape adaptation. It is challenging for unjammed sheet to achieve high material malleability and realize complex shapes with compound curvatures, as the sheet materials are not stretchable or can be only stretched in one direction. (Score: 7/10)	Medicinal plaster can be molded into any shape according to the contours of the body. (Score: 10/10)
Tunability	The bending modulus of the dual-faced fabric changes significantly with vacuum confining pressure. In addition, the bending modulus can be further changed by changing the loading surface. (Score: 10/10)	The bending modulus of the octahedral structured fabric can be changed significantly by various vacuum confining pressure. (Score: 7/10)	The bending modulus of the jamming sheet changes significantly with vacuum confining pressure. (Score: 7/10)	The mechanical properties of the unjammed sheet are fixed. (Score: 0/10)	The mechanical properties of medicinal plaster cannot be adjusted. (Score: 0/10)

* Present design

Section 8. Mechanical properties of MJF-printed PA12 bulk material

A preloading of around 5 N was set for all MJF-printed PA12 tensile specimens at horizontal build orientation. Uniaxial tensile tests with a displacement rate of 10 mm/min were performed on the tensile specimens. The uniaxial tensile test results of the tensile specimens are shown in **Figure S9** and Table S6. As observed, the stress–strain curves for the three samples closely overlap. The Young's modulus, ultimate tensile strength and elongation at break are 1404 MPa, 47.7 MPa and 20.7%, respectively. The mechanical properties of the MJF-printed PA12 bulk material obtained here are similar to those reported in previous studies [19, 20].

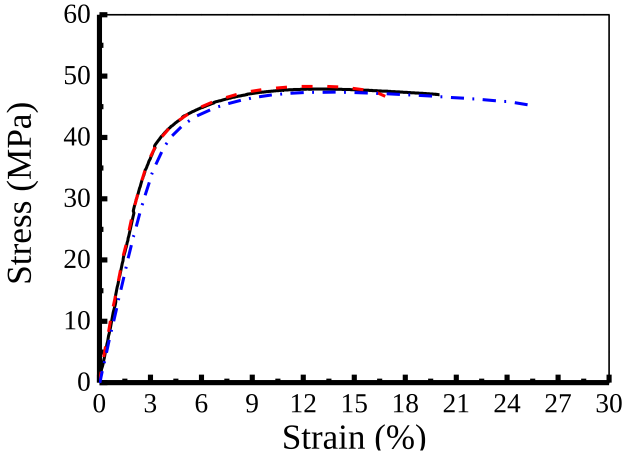

Figure S9. Tensile test results for MJF-printed PA12 bulk material.

Table S6. Mechanical properties of MJF-printed PA12 bulk material.

Mechanical property	Value
Young's modulus [MPa]	1404 ± 249
Ultimate tensile strength [MPa]	47.7 ± 0.5
Elongation at break [%]	20.7 ± 4.4

Note that defects are inevitable during the printing process, and our group has previously carried out a related study on the effect of void defects on the mechanical properties of the MJF-printed PA12 bulk material based on uniaxial tensile tests [21].

The results reveal that the expansion and coalescence of voids lead to crack initiation and propagation in the bulk material during uniaxial tensile deformation. In addition, our previous work has indicated that the porosity of MJF-printed bulk material can be decreased by optimizing the printing parameters, such as the build direction, power input, layer thickness, *etc*. [22]. Given the structural complexity of the dual-faced structured fabric, the effect of defects on the overall performance of the protection system has not been studied in the present work, but will be carried out as part of future work.

References

[1] Q. Li, I. Magkiriadis, J. J. Harrigan, *Journal of cellular plastics*, **2006**, *42* (5), 371.

[2] B. Hu, M. Li, J. Jiang, W. Zhai, *International Journal of Mechanical Sciences*, **2021**, *197*, 106324.

[3] X. Zhang, J. Yao, B. Liu, J. Yan, L. Lu, Y. Li, H. Gao, X. Li, *Nano letters*, **2018**, *18* (7), 4247.

[4] T. A. Schaedler, A. J. Jacobsen, A. Torrents, A. E. Sorensen, J. Lian, J. R. Greer, L. Valdevit, W. B. Carter, *Science*, **2011**, *334* (6058), 962.

[5] L. Salari-Sharif, T. A. Schaedler, L. Valdevit, *Journal of Materials Research*, **2014**, *29* (16), 1755.

[6] A. Aldoshan, S. Khanna, *Materials Science and Engineering: A*, **2017**, *689*, 17.

[7] S.-f. Fan, T. Zhang, Y. Kun, H.-j. Fang, H.-q. Xiong, Y.-l. Dai, D.-y. JIANG, H.-l. ZHU, Transactions of Nonferrous Metals Society of China, 2017, 27 (1), 117.

[8] S. Yuan, C. K. Chua, K. Zhou, *Advanced Materials Technologies*, **2019**, *4* (3), 1800419.

[9] L. R. Meza, S. Das, J. R. Greer, *Science*, **2014**, *345* (6202), 1322.

[10] L. Salari-Sharif, T. Schaedler, L. Valdevit, *Journal of Engineering Materials and Technology*, **2018**, *140* (3), 031003.

[11] W. Liu, H. Song, Z. Wang, J. Wang, C. Huang, *Materials & Design*, **2019**, *181*, 108065.

[12] X. Cao, D. Zhang, B. Liao, S. Fang, L. Liu, R. Gao, Y. Li, *Thin-Walled Structures*, **2020**, *157*, 107147.

[13] X. Zheng, W. Smith, J. Jackson, B. Moran, H. Cui, D. Chen, J. Ye, N. Fang, N. Rodriguez, T. Weisgraber, *Nature materials*, **2016**, *15* (10), 1100.

[14] X. Li, X. Yu, W. Zhai, *Advanced Materials* **2021**, *33* (44), 2104552.

[15] L. R. Meza, J. R. Greer, *Journal of materials science*, **2014**, *49* (6), 2496.

[16] Y. Wang, L. Li, D. Hofmann, J. E. Andrade, C. Daraio, *Nature*, **2021**, *596* (7871), 238.

[17] Y. S. Narang, J. J. Vlassak, R. D. Howe, *Advanced Functional Materials*, **2018**, *28* (17), 1707136.

[18] B. Szostakowski, P. Smitham, W. Khan, *The Open Orthopaedics Journal*, **2017**, *11*, 291.

[19] C. Cai, W. S. Tey, J. Chen, W. Zhu, X. Liu, T. Liu, L. Zhou, K. Zhou, *Journal of Materials Processing Technology*, **2021**, 288, 116882.

[20] K. Chen, Z.H. Koh, K.Q. Le, H.W.B. Teo, H. Zheng, J. Zeng, K. Zhou, H. Du, *Virtual and Physical Prototyping*, **2022**, 17(3), 631-648.

[21] K. Chen, Teo, H.W.B. Teo, Y. Tian, S. Wu, G. Kang, K. Zhou, J. Zeng, H. Du, *International Journal of Fatigue*, 2023, 107514.

[22] X. Liu, W. S. Tey, P. Tan, K.K. Leong, J. Chen, Y. Tian, A. Ong, L. Zhao, K. Zhou, *Virtual and Physical Prototyping*, **2022**, 17(3), 734-748.

Movie 1. Demonstration of how the dual-faced structured fabric can be easily stored and transported.

Movie 2. Compression response of the dual-faced structured fabric at a compression rate of 0.01 mm/s under the confining pressure of 60 kPa. The video is played at 30 times the normal speed.

Movie 3. Compression response of the octahedral structured fabric at a compression rate of 0.01 mm/s. The video is played at 15 times the normal speed.

Movie 4. Demonstration of the dual-faced structured fabric showing a significantly tunable bending stiffness.

Movie 5. Demonstration of the dual-faced structured fabric being used as a protective material for glassware.
